# Comparison of the Transdermal and Intravenous Administration of Buprenorphine in the Management of Intra- and Postoperative Pain in Dogs Undergoing a Unilateral Mastectomy

**DOI:** 10.3390/ani12243468

**Published:** 2022-12-08

**Authors:** Margherita Galosi, Alessandro Troisi, Pietro Toniolo, Luca Pennasilico, Vincenzo Cicirelli, Angela Palumbo Piccionello, Caterina Di Bella

**Affiliations:** 1 School of Bioscience and Veterinary Medicine, University of Camerino, 62024 Matelica, Italy; 2 Department of Veterinary Medicine (DMV), University of Bari Aldo Moro, 70010 Valenzano, Italy

**Keywords:** mastectomy, pain, buprenorphine, transdermal patches, dog

## Abstract

**Simple Summary:**

A mastectomy is the gold standard of treatment for mammary tumors in dogs; nevertheless, surgical stimuli cause extensive inflammation and moderate–severe perioperative pain. Opioids are the most widely used drugs in both human and veterinary medicine for the management of pain; however, their systemic administration is not free from side effects (nausea, vomiting, dysphoria, and cardiac and respiratory depression). The administration of analgesics through a transdermal route offers an interesting alternative for the maintenance of a stable and prolonged plasma concentration of the drug, therefore avoiding most of the drawbacks of their parenteral administration. The aim of this clinical study was to evaluate the intraoperative and postoperative analgesic efficacy of buprenorphine administered through transdermal patches compared to the intravenous route in dogs undergoing a monolateral mastectomy. The results of this study showed that the transdermal administration of buprenorphine could guarantee an analgesic quality equal to the injectable route. Furthermore, it must be considered that it would guarantee the continuous administration of an opioid while avoiding repeated injections (especially in fearful or aggressive dogs).

**Abstract:**

The aim of this prospective clinical study was to evaluate the effectiveness of transdermal patches of buprenorphine as an alternative route for the management of perioperative pain in dogs undergoing a unilateral mastectomy. Our hypothesis was that the transdermal route would allow the obtainment of an analgesic plan comparable to that of the injectable administration. Twelve dogs were divided in two groups. In the BupreP group (six dogs), buprenorphine patches were applied 40 h before the start of the surgery, guaranteeing a dosage of 5–6 μg/kg/h. In the BupreI group (six dogs), 20 μg/kg of buprenorphine was administered intravenously 30 min before the induction of anesthesia, and this was repeated every 6 h for 24 h. The main physiological parameters, sedation scores (0 = no sedation; 11 = deep sedation), and pain scores were monitored from 30 min before the surgery to 24 h after the end of anesthesia. All *p* values < 0.05 were defined as statistically significant. Thirty minutes before the surgery, the sedation scores were higher in BupreI (score = 10) compared to the BupreP group (score = 1). Moreover, during the mastectomy, the mean arterial pressure significantly increased in both groups even if nobody required additional analgesia. In the postoperative period, the pain scores did not show statistically significant differences between the two groups, maintaining values below the pain threshold at all times of the study. In conclusion, the transdermal administration of buprenorphine could guarantee an analgesic quality equal to that of the injectable route.

## 1. Introduction

Up to 70% of tumors in sexually intact female dogs are represented by mammary gland tumors. To date, the gold standard of treatment for most types of these tumors is surgery, with a simple or radical mastectomy, excluding inoperable highly metastatic diseases and most of the inflammatory mammary carcinomas [1]. A unilateral mastectomy can induce a significant pain stimulus which, if not correctly managed in the intra- and postoperative periods, can lead to hyperalgesia and peripheral sensitization [2,3]. Opioids are the most widely used drugs in both human and veterinary medicine for the management of moderate–severe pain; however, their systemic administration is not free from side effects, such as nausea, vomiting, dysphoria, and cardiac and respiratory depression [4]. Moreover, in veterinary medicine, it is also necessary to consider the difficulty of the repeated administration of injectable drugs (stressed, aggressive, and intractable patients) and the possibility of administering opioids exclusively during hospitalization [5]. The administration of analgesics via a transdermal route offers an interesting alternative for the maintenance of analgesia for long periods of time, therefore avoiding most of the drawbacks of chronic parenteral or oral administration [6]. The applicability and validity of the patches was already demonstrated in veterinary patients; the first successful studies on this topic concerned the use of lidocaine or fentanyl patches to provide analgesia for the management of acute pain in dogs [7,8,9]. In human medicine, the use of transdermal buprenorphine patches (TBP) has also been used successfully in both acute–chronic pain and offers several advantages: a low risk of respiratory depression, no pharmacological alterations in patients with renal failure, and less gastrointestinal adverse effects. However, in veterinary medicine, the available literature is still limited [10,11,12]. Buprenorphine is a partial agonist of µ-opioid receptors that is used for perioperative pain control as a part of multimodal analgesia [13]. The benefits of buprenorphine as an analgesic in dogs and cats include a long duration of action, a reduced risk of nausea and vomiting, and cardiovascular stability in healthy patients [14,15]. Therefore, the combination of a potent antinociceptive drug, an easy and painless route of administration, and a more stable and prolonged plasma concentration of the drug make TBPs a possible and useful alternative for the management of acute perioperative pain in dogs [5,16]. To the authors’ knowledge, there are no recent studies on the analgesic efficacy of buprenorphine patches in dogs for the management of acute moderate–severe intra- and postoperative pain. The aim of this clinical study was to evaluate the intraoperative and postoperative analgesic efficacies of buprenorphine administered with transdermal patches compared to an intravenous (IV) route in dogs undergoing a radical monolateral mastectomy. Our hypothesis was that the TBPs could guarantee an equal degree of analgesia compared to the intravenous administration, therefore representing a valid alternative to it. In order to validate our hypothesis, the main physiological parameters were monitored during the perioperative period. Moreover, the University of Melbourne Pain Scale (UMPS) and the Short Form of the Glasgow Composite Measure Pain Scale (CMPS-SF) were used to assess pain status during the postoperative period [17,18].

## 2. Materials and Methods

This prospective randomized, blinded clinical study was approved by the Ethics Committee for Clinical Study in Animal Patients of the University of Camerino with approval number Prot. 01/22, and informed owner consent was obtained for all dogs.

### 2.1. Animals

Twelve mixed-breed dogs (Table 1) with mammary gland tumors that were subjected to monolateral mastectomy, were included in this study. Patients with ASA physical status > II (according to the American Society of Anesthesiologists) or that were aggressive; pregnant; or with cardiac, renal, and hepatic diseases were excluded. In addition, dogs with pain resulting from other causes unrelated to the tumors or that were already undergoing any type of analgesic treatment were also excluded. The inclusion criteria consisted of subjects free from systemic diseases (ASA < III) with nonulcerated and nonmetastatic mammary tumors. All the aforementioned factors were evaluated through clinical examinations, FNAC (Fine Needle Aspiration Cytology), chest x-rays, abdominal ultrasounds, and hematobiochemical evaluations.

### 2.2. Anesthetic Protocol

All dogs were hospitalized 48 h before surgery, and fasting began 12 h earlier. Free access to water was maintained. The day of surgery, a cephalic vein was cannulated in order to administer drugs and fluids. Therefore, anesthesia was induced with 3–5 mg/kg of propofol (Proposure^®^, Boehringer Ingelheim Animal Health S.p.A) injected intravenously (IV) until adequate muscle relaxation was obtained (muscle relaxation of limbs, relaxation of jaws, and loss of pedal reflex). All subjects were intubated and connected to a circle rebreathing system, and general anesthesia was maintained with isoflurane (IsoFlo, Zoetis S.r.l) in pure oxygen (fractional inspired oxygen > 0.8). The peripheral venous catheter was connected to a volumetric pump (VP Volumetric Infusion Pump, Fresenius Kabi) to administer fluids (5 mL/kg/h, Ringer’s lactate, B. Braun) until the end of the procedure. Moreover, 60 min before the start of surgery, amoxicillin–clavulanic acid (20 mg/kg, Synulox, Zoetis S.r.l.) was injected subcutaneously (SC). All patients included in this study were maintained at spontaneous breathing. A multiparametric monitor (BeneView T8, Mindray Medical S.r.l) was used to assess the main parameters. Moreover, the following cardiovascular and respiratory data were manually collected every five minutes during the entire procedure: heart rate (HR; beats/minutes); systolic, mean, and diastolic blood pressure (SAP, MAP, DAP; mmHg), respectively; peripheral capillary oxygen—hemoglobin saturation (SpO_2_; %); respiratory rate (RR; breath/minute); end-tidal concentration of carbon dioxide (EtCO_2_; mmHg); end-tidal concentration of isoflurane (Et_Iso_; %); inspired fraction of isoflurane (Fi_Iso_; %); minimum alveolar concentration of isoflurane (MAC; %); and temperature (T, ◦C). In case of intraoperative hypotension events, (MAP < 60 mmHg and/or DAP < 40 mmHg) for two consecutive readings (3 min interval), a bolus of colloids (3 mL/kg, Gelofusine, B. Braun) was administered through IV. If the hypotensive status persisted for another ten minutes after the bolus, a continuous infusion of dopamine (10 µg/kg/min, Dopamine, PH&T S.p.A) was started. Moreover, in case of bradycardic events (HR < 40 beats/minute) for more than 2 min, atropine sulphate was administered (0.02 mg/kg, Atropine Sulfate, Fatro S.p.A) through IV [19]. At the end of the surgery, time of extubation, duration of anesthesia (from the start of administration of isoflurane to the discontinuation of this), and duration of surgery (from the cut of the skin to the apposition of the last suture) were registered. During the recovery phase, hypothermia (T < 37 °C) and low SpO_2_ (< 95%) were managed with suitable thermal support and supplementary oxygen (face mask or flow by), respectively. In addition, all dogs received non-steroidal anti-inflammatory drugs (carprofen 4 mg/kg, SC; Rimadyl, Zoetis S.r.l) 1 h after the surgery. All procedures were performed by the same experienced surgeon.

### 2.3. Study Protocol

**Figure 1:**
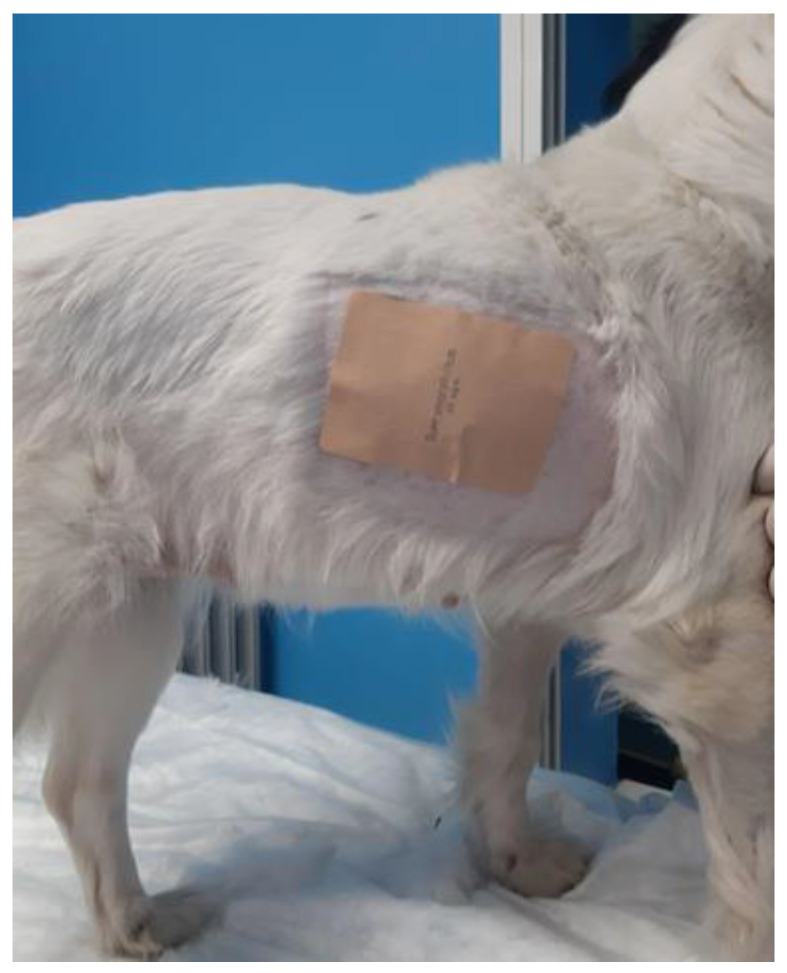
Placement of the TBP on the thorax after appropriate shearing and washing of the area.

Dogs were randomly allocated to one of two groups using a random number generator (Microsoft^®^ Excel^®^, Microsoft 365 MSO 2021):

BupreP group (6 dogs): The TBPs were applicated 40 h before the surgery in order to achieve an adequate plasmatic concentration of the drug as previously described [16]. For each dog, one or more patches were chosen, ensuring a standardized dosage [4]. Patches releasing from 5, 10, 20, 35, 52.5, and up to 70 mcg/h (Busette, Torrinomedica; TransTec, Grunenthal S.r.l). were used based on patient weight. No patches were cut, and the dosage was calculated, keeping a range between 5 and 6 μg/kg/h by applying one or more patches based on the weight of the dog (e.g., weight 3.5 kg = 17.5–21 μg/h = application of two patches that release 10 μg/h). Patches were applied monolaterally or bilaterally in the upper third of the thorax based on the number of patches used and the size of the dog. The area was shaved, cleaned with physiological solution (NaCl 0.9%, B. Braun), and dried with swabs to ensure the perfect adherence of the patches (Figure 1). After that, thorax was covered with a soft elastic bandage to avoid episodes of lambing or scratching that could cause the patches’ detachment.

BupreI group (6 dogs): After the placement of the venous catheter, 30 min before the induction of general anesthesia, 20 μg/kg of buprenorphine (Buprefelican; Le Vet Beheer^®^, Dechra Veterinary Products S.r.l) was administered through IV. Moreover, dogs received the same dose of buprenorphine every 6 h for the next 24 h.

#### 2.3.1. Intraoperative Assessment

Fifteen minutes before the induction, the main physiological parameters (HR, RR, T, and MAP) and the sedation scores (SS) were registered in both groups. The SS was obtained through the composite simple descriptive sedation score [20] with the score ranging from 0 (lack of sedation) to 15 (maximum sedation). The intraoperative data (HR, RR, MAP, T, and MAC) were recorded 10 min prior to the start of surgery (BASE) during the skin incision (SKIN), the mammal line traction and dissection (MAST), and the suture of the subcutaneous plane (SUTURE). During the procedure, an increase in HR or MAP greater than 20% from BASE for more than 1 min was considered a significant nociceptive autonomic response to surgical stimulation; thus, patients were withdrawn from the study, and a constant rate infusion (CRI) of lidocaine was started (30–50 μg/kg/h) [21]. Surgery duration was measured from the first incision until the end of skin suture, while anesthesia duration was defined as the total time of isoflurane administration. Extubation time was also registered.

#### 2.3.2. Postoperative Assessment

A blinded trained evaluator recorded HR, RR, MAP, and T at 2, 4, 6, 8, 10, 15, 20, and 24 h post extubation (T2, T4, T6, T8, T10, T15, T20, T24, respectively). Moreover, the SF-CMPS and the UMPS were used to assess pain during the recovery (17,18). If the SF-CMPS and/or UMPS showed a score greater than 4/20 and 6/27, respectively, a bolus of buprenorphine (10 μg/Kg) was administered through IV. Also, if dogs showed a persistent status of pain, they were excluded, and a new analgesic plan was chosen according to clinical requirements. The times and the amount of the supplemental analgesia were recorded. At each time of study, the appetite and the accomplishment of the great organic functions (urination and defecation) were also recorded.

### 2.4. Statistical Analysis

A sample size calculation was performed considering previous data in dogs [4]. Power calculation was conducted for a two-tailed *t*-test considering the mean postoperative HR as the reference parameter, with a power of 0.95 and an alpha error of 0.05 (G*Power Version 3.1.9.3). The test suggested that a minimum of 6 dogs could be sufficient to detect significant differences, with effect size of 4.75 [22]. Statistical analysis was performed using MedCalc software 9.0 (MedCalc version 9.2.10; MedCalc Software). Data were tested for normality with Shapiro–Wilk test and were summarized as mean ± standard deviation or median (minimum–maximum). Parametric data were analyzed with one-way ANOVA test to perform a comparison between groups. ANOVA for repeated measures was used to compare the study times within groups. Nonparametric data were analyzed with Kruskal–Wallis test and Dunn post hoc test to obtain a comparison between the three groups. Friedman test was used to compare the study times within each group. A *p* value < 0.05 was considered statistically significant.

## 3. Results

**Figure 2:**
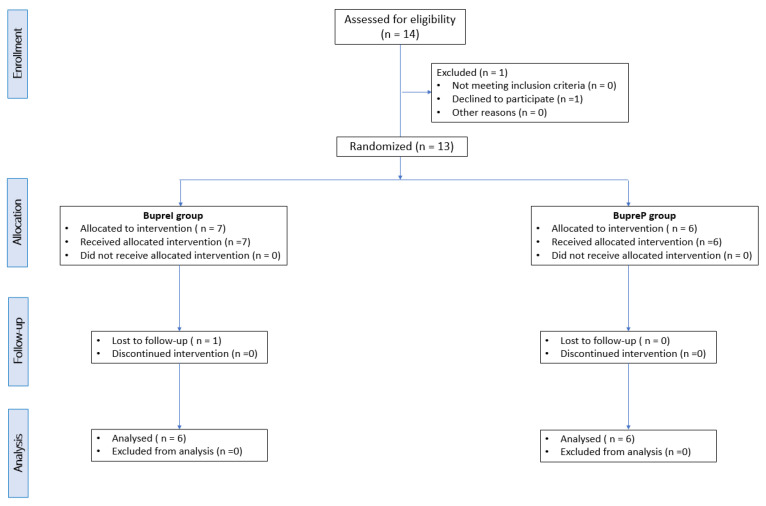
Consolidated Standards of Reporting Trials (CONSORT) flow diagram for dogs included in the study.

A total of 14 dogs were assessed for eligibility. Of these, two dogs were excluded (n. 1 = the owners refused to participate; n. 1 = request for rescue analgesia during surgery). The remaining 12 dogs were successfully included. The manuscript conformed to the Consolidated Standards of Reporting Trials (CONSORT) Statement 2010 for reporting randomized clinical trials [23] (Figure 2). There were no significant differences between the groups in age (BupreP = 10 ± 0.8; BupreI = 10.75 ± 2.6 years), weight (BupreP = 13.16 ± 9.4; BupreI = 11.95 ± 9.7 kg), the duration of the surgery (BupreP = 85.5 ± 27.2; BupreI = 74.7 ± 3.2 min), and the duration of anesthesia (BupreP = 106.5 ± 29.3; BupreI = 105.7 ± 8.5 min). Instead, the SS showed significant differences between two groups in the study (BupreP = 1 (1–2); BupreI = 10 (7–11)) (Table 2). In BupreP, the mean of the total dose of buprenorphine administered was 5.39 ± 0.25 μg/kg/h.

### 3.1. Intraoperative Assessment

The MAP increased in both groups at the MAST time (BupreP = 77.5 ± 16.2; BupreI = 78 ± 10.7 mmHg) compared to the BASE (BupreP = 67.5 ± 9.1; BupreI = 60.8 ± 6.5 mmHg). Moreover, in BupreP, it was also significantly higher at the SUTURE time (BupreP = 73.7 ± 14.8 mmHg) than the BASE. Anyway, the MAP reached the increase of 20% required for the administration of analgesic drugs in one subject in the BupreI group. The dog received rescue analgesia, and it was excluded. The other parameters analyzed did not show statistically significant differences (Table 3).

### 3.2. Postoperative Assessment

**Figure 3:**
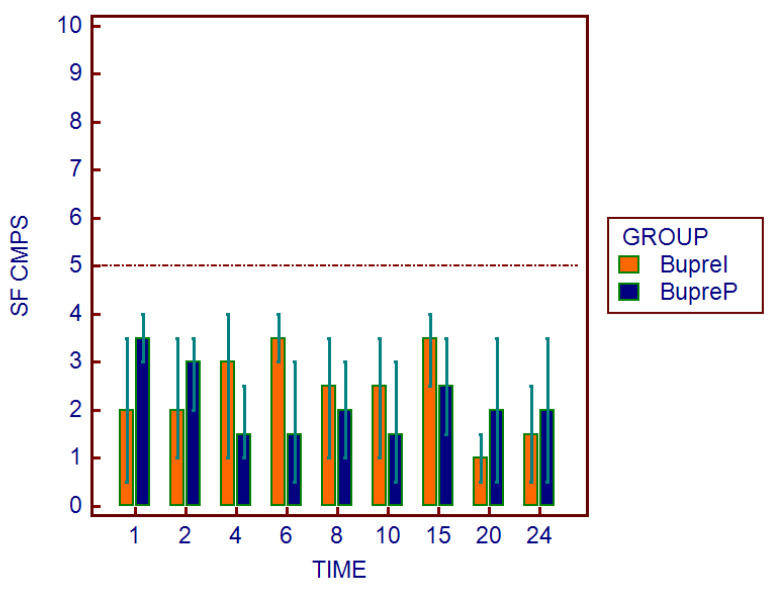
Graphical representation of the SF-CMPS scores in the two study groups at different times (1, 2, 4, 6, 8, 10, 15, 20, and 24 h after the extubation). The dotted line indicates the maximum score level above which dogs required supplemental analgesia.

**Figure 4:**
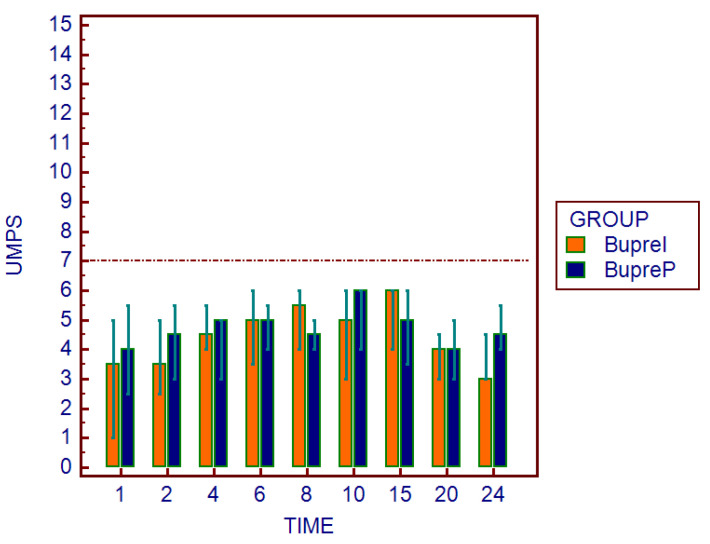
Graphical representation of the UMPS scores in the two study groups at different times (1, 2, 4, 6, 8, 10, 15, 20, and 24 h after the extubation). The dotted line indicates the maximum score level above which dogs required supplemental analgesia.

The physiological parameters did not show statistically significant differences. Similarly, as regards the assessment of the multiparameter pain scales, there were no significant differences between the two groups at all study times. Moreover, no subject reached a score of 5/20 on the SF-CMPS (Figure 3) and/or 7/27 on the UMPS (Figure 4), and the administration of a rescue analgesia in the postoperative period was not necessary in either group (Table 4). A skin reaction (redness) was found in the area of the application of the patch on one subject after the removal of the patches.

## 4. Discussion

Based on the results obtained in this study, the intravenous and transdermal administration of buprenorphine elicited the same efficacy in the management of intra- and postoperative pain in dogs undergoing a unilateral mastectomy. In this clinical study, unlike those previously performed in veterinary medicine, the authors chose to select the buprenorphine patches to be applied based on the weight of dogs. Moll et al. demonstrated that the 70 µg/h patch dose of buprenorphine was enough to achieve a good analgesic plan in dogs with an average weight of 12.67 ± 1.58 kg undergoing ovariohysterectomy [4]. Based on these results, it was decided to identify a dosage of 5–6 µg/kg/h as sufficient for the obtainment of good analgesia in our patients. Regarding the time interval between the application of the patches and the start of the surgical procedure, the indications provided in the literature were adopted. It was showed that the plasma concentration of buprenorphine, after the application of the patch, gradually increased over the next 36–48 h, maintaining a constant plasma plateau for up to 108 h [15,16].

In this study, during the preoperative evaluations, the sedation scores, evaluated 15 min before surgery, were significantly higher in the group that received injectable buprenorphine. These results were probably due to the achievement of a high plasma concentration of the drug in a short time and, therefore, to a greater diffusion gradient between the plasma and central nervous system. In contrast, the slow and gradual release of buprenorphine through the patch did not cause sedation in dogs [16,24].

During the surgery and specifically at the MAST time (the dissection and traction of the mammal tissues), one patient required rescue analgesia and was withdrawn from the study. In both groups, the MAP was significantly higher than it was at baseline. This transient peak could be associated with the highly nociceptive stimulation created by the traction of the tissue itself [3]. A mastectomy is a painful procedure, and, probably, buprenorphine alone (whether given by injection or transdermal patches) was not sufficient in managing the nociceptive stimulus. This led us to believe that the use of buprenorphine patches would be more suitable in the management of postoperative pain rather than acute intraoperative pain where the choice of a multimodal analgesic plan could be more effective (e.g., adding locoregional anesthesia) [2,25,26].

Otherwise, regarding the monitoring of postoperative pain obtained through the SF-CMPS and UMPS, no statistically significant differences were found between the two groups. Furthermore, no subject achieved a score such as to require rescue analgesia. Therefore, the analgesic efficacy obtained through the transdermal route seemed to be as effective as that of the injectable route. In human medicine, the efficacy of buprenorphine transdermal patches in pain management after a gynecological surgery has already been widely demonstrated [27,28,29]. However, in veterinary medicine, there are currently only a few studies demonstrating its efficacy. Piper et al., in an experimental model of acute pain in dogs, showed the antinociceptive action of buprenorphine administered via a patch approximately 36 h after its application [16]. Subsequently, Moll et al., in agreement with our results, compared the efficacy of TBPs versus the subcutaneous administration of 20 µg/kg of buprenorphine in dogs undergoing an ovariohysterectomy, therefore demonstrating its efficacy [4]. However, it is the authors’ opinion that the advantages and disadvantages of this alternative route of administration should be considered.

The advantages include the possibility of reducing the pain stimulation caused by the administration of analgesic drugs through an injectable route. This aspect is even more significant in the case of aggressive, fearful, and uncooperative patients in which manipulations are an additional stress factor [30]. The possibility of guaranteeing a good analgesic plan, therefore reducing stress, offered a greater degree of welfare in our patients. Another factor to consider is the maintenance of a constant plasma concentration of the drug. The use of repeated boluses does not allow the maintenance a stable plasmatic concentration, which could lead to an incomplete analgesic plan. On the other hand, after the application of the patch, plasma buprenorphine concentrations increased slowly during the first 36–48 h and remained in a steady-state period for 108 h, ensuring continuous analgesia for about 5 days [11,15]. Unfortunately, there are no studies evaluating the timing of the excretion of buprenorphine after patch removal in dogs. Murrell et al. observed a plasma peak in cats two hours after the removal of the patch (most likely due to the reabsorption of the drug that was deposited in the skin) as well as the drug’s presence in the blood even 24 h after the removal of the patch [24]. However, this is a single study including six cats, so further research is needed to investigate the topic. Another aspect to consider is that buprenorphine has a high potency; it is approximately 30 times more potent than morphine. Its high-affinity ligand binding and slow receptor dissociation cause buprenorphine to have a high µ receptor occupancy, even at low doses. For this reason, the interaction between buprenorphine and other pure agonists is not yet clear. Therefore, if the analgesic plan provided by the patch is not sufficient, the efficacy of other opioids, such as methadone or morphine, could be unpredictable [31,32,33]. Moreover, another aspect to consider is the slow achievement of an adequate plasma plateau after the application of the patches (over 36 h). This disadvantage makes TBPs unsuitable for the immediate and rapid treatment of acute pain [15,16,34]. In this study, after removing the patch, the presence of skin redness was observed in one subject. Temporary skin irritation, due to individual sensitivity to the active substance or to the patch matrix itself, is rare and has also been reported in other studies involving the use of transdermal patches in dogs [7,35]. In the present study, the skin reaction that was found healed without treatment in 24 h.

The limitations of our study to consider are the small sample size and the absence of data relating to the duration of the analgesic effect after the removal of the patch. It could be interesting to evaluate the maximum release time of buprenorphine from the patch and the excretion times of the drug in dogs.

## 5. Conclusions

The transdermic patches of buprenorphine seemed to have a comparable analgesic efficacy to that of the injectable route, showing a good alternative for the management of perioperative pain in dogs. Furthermore, compared to the intravenous route, TBPs provided a prolonged and constant analgesic treatment, therefore reducing the stress of multiple manipulations. However, further clinical and pharmacokinetic/pharmacodynamic studies are required.

## Figures and Tables

**Table 1:** List of dog breeds included in the clinical study.

N°	Breed
**7**	Half-breed
**2**	Cocker Spaniel
**1**	Jack Russel Terrier
**1**	Chihuahua
**1**	Maremma Sheep

**Table 2:** Mean ± SD of weight, age, and duration of surgery and anesthesia in both groups. The sedation score (SS) was compared between the two groups 15 min before the induction (15 min after the administration of buprenorphine in BupreI group). * *p* < 0.05.

	Weight (kg)	Age (Years)	Surgery (Minutes)	Anesthesia (Minutes)	SS
BupreP	13.16 ± 9.4	10 ± 0.8	85.5 ± 27.2	106.5 ± 29.3	1 (1–2)
BupreI	11.95 ± 9.7	10.75 ± 2.6	74.7 ± 3.2	105.7 ± 8.5	10 (7–11) *

**Table 3:** Mean ± SD of the intraoperative parameters ten minutes before the start of surgery (BASE) and during the incision of the skin (SKIN), the mammal line traction and dissection (MAST), and the suture of the subcutaneous plane (SUTURE). ^a^ *p* < 0.05 compared with BASE.

Parameter	Group	Base	Skin	Mast	Suture
HR (beat/min)	BupreP	106.5 ± 30 106 ± 41.9	100.5 ± 24.4 102 ± 37.7	114.5 ± 15.9 95.2 ± 32.4	106.2 ± 11.6 106.8 ± 34.07
	BupreI				
RR (breath/min)	BupreP	6.2 ± 2.5 12.8 ± 6.3	6 ± 1.41 9.6 ± 4.5	9.2 ± 4.1 14.2 ± 7.5	8 ± 1.6 9.2 ± 4.4
	BupreI				
MAP (mmHg)	BupreP	67.5 ± 9.1 60.8 ± 6.5	70.25 ± 6.7 64.8 ± 11.5	77.5 ± 16.2 ^a^ 78 ± 10.7 ^a^	73.7 ± 14.8 ^a^ 72.2 ± 7.5
	BupreI				
T (°C)	BupreP	35.9 ± 1.1 36.7 ± 0.65	35.7 ± 1.1 36.4 ± 0.8	34.9 ± 0.9 35.8 ± 1.2	34.6 ± 0.7 35.4 ± 0.9
	BupreI				
MAC (%)	BupreP	1.1 ± 0.2 0.8 ± 0.1	1.1 ± 0.2 1.02 ± 0.1	1.2 ± 0.2 1.02 ± 0.12	1.1 ± 0.2 1.09 ± 0.07
	BupreI				

**Table 4:** Mean ± SD of physiological parameters and median (min–max) of the pain scores at 2, 4, 6, 8, 10, 15, 20, and 24 h after extubation (T2, T4, T6, T8, T10, T15, T20, and T24, respectively).

Parameter	Group	T2	T4	T6	T8	T10	T15	T20	T24
HR (b/min)	BupreP	91 ± 38.2 87 ± 22.7	91 ± 25.1 84 ± 11.7	83 ± 19.9 82 ± 11.5	96.2 ± 28 83.5 ± 12.2	94 ± 20.7 95 ± 3.8	91 ± 36.1 93.7 ± 21.8	102.7 ± 40.4 85 ± 5	94.5 ± 32.1 85.7 ± 11.3
	BupreI								
RR (breath/min)	BupreP	43 ± 24.9 21 ± 5	46 ± 34.6 32 ± 12.6	37 ± 22.9 25 ± 7.5	38 ± 25.4 20 ± 5.6	44 ± 20.9 24 ± 12.6	41.5 ± 26.8 25 ± 2	25 ± 10.5 24 ± 10.8	36 ± 23.7 28 ± 10.3
	BupreI								
MAP (mmHg)	BupreP	102.6 ± 11 112.5 ± 32.3	94.5 ± 26.4 100.2 ± 20	92.5 ± 21.2 115 ± 33.1	95.7 ± 29.3 102.5 ± 16.3	94 ± 15.6 94.7 ± 27.8	95.7 ± 16.4 106 ± 15.8	109.2 ± 14.4 111.2 ± 10.3	113.3 ± 2.8 112.2 ± 19.5
	BupreI								
UMPS	BupreP	4.5 (2–6) 3.5 (2–6)	5 (1–5) 4.5 (4–6)	5.5 (3–6) 5 (3–6)	4.5 (4–5) 5.5 (3–6)	6 (2–6) 5.5 (2–6)	5 (3–6) 6.5 (2–6)	4 (2–6) 4 (2–5)	4.5 (4–6) 3 (3–6)
	BupreI								
SF-CMPS	BupreP	3 (1–4) 2 (1–4)	1.5 (1–3) 3 (0–4)	1.5 (0–4) 3.5 (3–4)	2 (0–4) 2.5 (0–4)	1.5 (0–4) 2.5 (0–4)	2.5 (1–4) 3.5 (2–4)	2 (0–4) 1 (0–2)	2 (0–4) 1.5 (0–3)
	BupreI								

## Data Availability

The data presented in this study are available on request from the corresponding author.
